# Traumatic brain injury-induced disruption of the circadian clock

**DOI:** 10.1007/s00109-024-02416-w

**Published:** 2024-01-29

**Authors:** Lu-Ting Kuo, Hsueh-Yi Lu, Yi-Hsing Chen

**Affiliations:** 1 Division of Neurosurgery, Department of Surgery, National Taiwan University Hospital, 7 Chun-Shan South Road, Taipei, 100 Taiwan; 2 Division of Neurosurgery, Department of Surgery, National Taiwan University Hospital, Yun-Lin Branch, Douliu, Yunlin 640 Taiwan; 3 Department of Industrial Engineering and Management, National Yunlin University of Science and Technology, Douliu, Yunlin 640 Taiwan

**Keywords:** Cosinor analysis, Circadian gene, Temperature, Traumatic brain injury

## Abstract

**Abstract:**

Disturbances in the circadian rhythm have been reported in patients following traumatic brain injury (TBI). However, the rhythmic expression of circadian genes in peripheral blood leukocytes (PBL) following TBI has not yet been studied. The messenger ribonucleic acid (mRNA) expression of period 1 (*Per1*), *Per2*, *Per3*, cryptochrome 1 (*Cry1*), *Cry2*, brain and muscle aryl hydrocarbon receptor nuclear translocator-like 1 (*Bmal1*), and circadian locomotor output cycles kaput (*Clock*) was quantified in PBLs from sham-operated rats and rats with acute subdural hematoma (ASDH) over a 48-h period. The rectal temperature of the animals was measured every 4 h over 2 days. The mesor, rhythm, amplitude, and acrophase were estimated using cosinor analysis. Cosinor analysis revealed that *Per2*, *Cry1*, and *Bmal1* mRNAs were rhythmically expressed in the PBLs of sham-operated rats. In contrast, fluctuations in rhythmic expression were not observed following ASDH. The rectal temperature of sham-operated rats also exhibited rhythmicity. ASDH rats had a disrupted rectal temperature rhythm, a diminished amplitude, and an acrophase shift. TBI with ASDH results in dysregulated expression of some circadian genes and changes in body temperature rhythm. Further research is required to understand the pathophysiology of altered circadian networks following TBI.

**Key messages:**

- First to investigate the mRNA expression of circadian genes in PBLs of ASDH rats.
- ASDH rats had disrupted rhythmicity of Per2, Cry1, and Bmal1 mRNA expression.
- Cosinor analysis showed that ASDH rats had a disrupted rectal temperature rhythm.

## Introduction

Acute subdural hematoma (ASDH) is a common consequence of head injury in humans, characterized by the accumulation of blood between the dura and arachnoid membrane. Although there have been many relevant advances in emergency services, including multimodality neuromonitoring and neurointensive care, ASDH remains associated with high morbidity and mortality. Even if patients survive without major neurological deficits, they may encounter sleep–wake disturbances, particularly fatigue, hypersomnia, and insomnia [1–3].

Circadian rhythms are cycles lasting approximately 24 h and entail various physiological and molecular changes. Rhythms are endogenously generated in response to light, darkness, and other environmental cues [4, 5]. Circadian fluctuations play critical roles in the regulation of biological processes within the body, including the sleep–wake cycle, body temperature, eating habits, cardiovascular function, hormonal rhythms, and metabolism [6, 7]. The regulation of biological rhythms aids organisms to anticipate and adapt to environmental changes. The timings of mammalian circadian rhythms are classically inferred based on core body temperature (cBT) changes, melatonin secretion by the pineal gland, and blood cortisol levels. The primary circadian clock is in the suprachiasmatic nucleus (SCN), a bilateral structure located in the hypothalamus.

A subset of neurons within the SCN is the basis for circadian clock resetting through light entrainment and melatonin secretion. The neurons are sensitive to light signals transduced from the retina via the retinohypothalamic tract. External cues, such as the light–dark cycle, environmental temperature, and timing of food intake, can entrain these internal biological rhythms [7]. The circadian fluctuations are regulated through rhythmic gene expression within a complex neural regulatory network involving transcription and translation feedback loops, which ultimately cause oscillation [8]. In turn, output from the SCN controls the circadian rhythm throughout the body via the regulation of circadian gene expression in peripheral tissues and autonomic nervous system activity [8, 9]. Autonomous clocks exist in all peripheral tissues and are driven and synchronized by the SCN via circadian output pathways [10]. Circadian gene expression within the SCN governs the rhythms of various cellular metabolic processes, neuronal firing, and neuropeptide secretion, which ultimately manifest in physiological and behavioral rhythms [9, 11, 12]. A previous study demonstrated that 40.7% of patients with moderate-to-severe traumatic brain injury (TBI) exhibit disturbed circadian rhythms of brain temperature with a diminished amplitude in the first 72 h following operation [13]. However, the effects of TBI on the regulation of circadian rhythm-related gene expression in peripheral tissues have not yet been investigated in humans or animal models.

In this study, we analyzed the messenger ribonucleic acid (mRNA) expression dynamics of seven circadian rhythm-related genes in peripheral blood leukocytes (PBLs) from sham-operated and ASDH rats. The rhythmicity of cBT of sham-operated and ASDH rats was also analyzed. Finally, cosinor analysis was used to analyze the rhythmicity, mesor, amplitude, and acrophase of mRNA expression and cBT.

## Materials and methods

### Ethics statement

This study complied with the ARRIVE guidelines and was carried out in accordance with the U.K. Animals (Scientific Procedures) Act, 1986 and associated guidelines, EU Directive 2010/63/EU for animal experiments, or the National Research Council’s Guide for the Care and Use of Laboratory Animals. The study was approved by the Institutional Animal Care and Use Committee of National Taiwan University, College of Medicine (approval no. 20120485; date of approval: April 20, 2012).

### General preparation

Fourteen 8-week-old male Sprague–Dawley rats, weighing 190–210 g, were used in the study. Rats were allowed to acclimate to the animal room lighting conditions for 2 weeks prior to surgical procedures. The room temperature and relative humidity were maintained at 22 ± 2 °C and 50% ± 20%, respectively. The lighting conditions in the animal room were lights on from 07:00 to 19:00, with a 12:12 h cycle. The animals received water and food ad libitum prior to the experiments. Animals were randomized to the sham-operated group (*n* = 7) and ASDH group (*n* = 7). Data were analyzed between these groups.

### Induction of subdural hematoma

Following general preparation, animals in the ASDH group (*n* = 7) were placed in a prone position. Rats were then anesthetized with 2.5% isoflurane. Thereafter, an 8-mm sagittal scalp incision was made. A 3-mm frontal burr hole was drilled into the right frontal region, 3 mm from the sagittal suture and 1 mm anterior of the coronal suture, using surgical loupes. The dura was incised with a 26-gauge needle and a Codman microsensor (Codman, Raynham, MA, USA), connected to the Codman intracranial pressure (ICP) monitor, was inserted into the subdural space together with a 26-gauge L-shaped needle. The hole was secured with bone wax. This model is a modified version of the established models [14–16]. Nonheparinized venous blood was obtained from the tail vein of rats, and 0.06–0.1 mL was slowly injected (> 1 min) into the subdural space through the needle via the burr hole until the ICP was 22–25 mmHg. Next, the ICP catheter was removed, and subsequently, the dural opening was sealed with gelfoam, whereas the burr hole was sealed with bone wax. The scalp incision was closed using nylon sutures. Rats were returned to housing conditions under a 12-h light–dark cycle, and ad libitum oral intake was resumed postoperatively. Considering the effects of the day-night cycle on the study of circadian rhythm, all surgical procedures were completed by 5 AM. Blood sampling for mRNA expression analysis and cBT measurements was performed from 9 AM on the same day. Blood for analysis was collected from the tail veins every 4 h for 48 h. To analyze the effects of ASDH on cBT regulation, the rectal temperature of the animals was measured at 4-h intervals using a temperature probe. In sham-operated animals, no injection into the subdural space was made after placement of the ICP catheter and needle.

At 48 h, rats were euthanized via intraperitoneal injection of a lethal dose of pentobarbital. None of the animals exhibited obvious hemiparesis or other focal neurological deficits. No seizures were observed. Considering that ICP-induced brain injury with edema may exacerbate in the first few days after ASDH, a two-day survival time was chosen for the present experiments.

### Sample collection and mRNA extraction

Local anesthetic cream was applied on the surface of the tail 30 min before the experiment to minimize the effects of anesthesia on circadian gene expression. Peripheral blood samples of 100 µl each were collected from the tail veins of rats at 4 h intervals over a span of 48 h following the surgical procedure. These samples were collected to analyze the 24-h gene expression rhythms. For overnight data collection time points, tail vein blood sampling was conducted in a darkened room, with the only illumination being provided by red light. mRNA transcript measurements at 12 time points provided sufficient data for the estimation of cosinor parameters [17]. Total ribonucleic acid (RNA) was extracted from samples using the QIAamp RNA Blood Mini Kit with on-column deoxyribonuclease (DNase) treatment of RNA samples (Qiagen, Valencia, CA, USA) under strict RNAse-free conditions.

### cDNA synthesis and quantitative PCR

Up to 1 µg RNA was reverse-transcribed into complementary DNA (cDNA) using the Omniscript Reverse Transcription Kit (Qiagen) and 10 µM oligo-dT primers (Applied Biosystems, Waltham, MA, USA) in accordance with the manufacturer’s instructions. The 20-µL reaction volume containing the completed first-strand cDNA synthesis reaction was diluted to 50 µL, and 1 µL of this dilution was used for each quantitative polymerase chain reaction (PCR). PCRs were performed on an Illumina Eco™ Real-Time PCR System (Illumina, San Diego, CA, USA) with the following reaction conditions: initial denaturation at 95 °C for 10 min, 40 cycles with 10 s denaturation at 94 °C, and 30 s annealing at 60 °C. mRNA expression measurements were performed in triplicate, and the average was calculated. Relative expression levels for the means of the triplicate experiments for circadian genes were normalized to those of β-actin as an internal control, and the relative threshold cycle (∆Ct) was obtained. The 2^−ΔΔCt^ method was used to analyze mRNA expression [18, 19]. The circadian genes analyzed were period 1 (*Per1*), *Per2*, *Per3*, cryptochrome 1 (*Cry1*), *Cry2*, circadian locomotor output cycles kaput (*Clock*), and brain and muscle aryl hydrocarbon receptor nuclear translocator-like 1 (*Bmal1*), and the primers employed are listed in Table 1 (*Per3*, *Cry2* [20]; *Per1*, *Per2*, *Cry1*, *Clock* [21]; *Bmal1* [22]). The primers for β-actin were designed using Primer3 (version 4.0; https://bioinfo.ut.ee/primer3/) and Primer-BLAST (Basic Local Alignment Search Tool, NCBI*)*. The mRNA expression of clock genes was quantified using β-actin as the reference gene, which has been used as a housekeeping gene for circadian rhythm studies in several tissues [23–25]. The circadian oscillations of mRNA expression, mesor (mean level of mRNA oscillations), amplitude of the rhythm (used to measure half of the difference between the lowest and highest levels of mRNA expression), and acrophase (the time when mRNA expression reaches its peak during the day), were determined.

**Table 1:** Primer sequences for real-time polymerase chain reaction (PCR)

**Gene**	**Accession no**	**Primer sequence**	**Product size (bp)**
*Per1*	AB002108	Forward 5′-CGCACTTCGGGAGCTCAAACTTC-3′	169
		Reverse 5′-GTCCATGGCACAGGGCTCACC-3′	
*Per2*	NM_031678	Forward 5′-CACGCAACGGG GAGTACATCACAC-3′	142
		Reverse 5′-CAAGGGGAGGCTGCGAACACAT-3′	
*Per3*	XM_039110819.1	Forward 5′-GCAGGGCATTTGCGTGGA-3′	115
		Reverse 5′-GTGTCTCTCGGCTGGGAAATAC-3′	
*Cry1*	NM_198750	Forward 5′-GTGGTGGCGGAAACTGCTCTC-3′	152
		Reverse 5′-ACTCTGTGCGTCCTCTTCCTGA-3′	
*Cry2*	NM_133405.2	Forward 5′-GTGCTTTCTTCCAACAGTTCTTCC-3′	94
		Reverse 5-GGCAGGTATCGCCGGATGTA-3′	
*Bmal1*	XM_039109788	Forward 5′-TGGACTGCAACCGCAAGAG-3′	154
		Reverse 5′-CCTTCCATGAGGGTCATCTTTG-3′	
*Clock*	NM_021856	Forward 5′-TTCGATCACAGCCCAACTCC-3′	163
		Reverse 5′-ACCTCCGCTGTGTCATCTTCTC-3′	
*β-actin*	NM_031144.3	Forward 5′-ACCGAGCGTGGCTACAGCTTCACC-3′	107
		Reverse 5′-GTGGCCATCTCTTGCTCGAAGTCT-3′	

*Per1* period 1, *Per2* period 2, *Per3* period 3, *Cry1* cryptochrome 1, *Cry2* cryptochrome 2, *Bmal1* brain and muscle aryl hydrocarbon receptor nuclear translocator-like 1, *Clock* circadian locomotor output cycles kaput

### cBT analysis

The rectal temperature of the animals was measured using an anal probe every 4 h over 2 days, beginning from 9 AM on the day of operation. This time point was 4 h after the operation to ensure the stabilization of body temperature after general anesthesia and potential postoperative hypothermia. Temperature measurements at eight-time points provided sufficient temperature records for a better estimation of the cosinor parameters [17]. The following characteristics of rectal temperature were analyzed: circadian oscillations of temperature, mesor, amplitude of the temperature rhythm, and acrophase.

### Cosinor analysis

The circadian rhythm of mRNA expression and rectal temperature was analyzed using the cosinor method, which is the most commonly used approach for analyzing the diurnal pattern of biological rhythms [17, 26, 27]. Cosinor analysis is a nonlinear model that fits the data to a 24-h cosine curve, with estimates of rhythm, including mesor, amplitude, and acrophase [27]. An online platform (https://cosinor.online/app/cosinor.php) was used in this study [28]. If the characteristics of the data analyzed fit the cosine curve with *P* < 0.05, a circadian rhythm was confirmed.

## Results

### mRNA expression of circadian genes

**Fig. 1:**
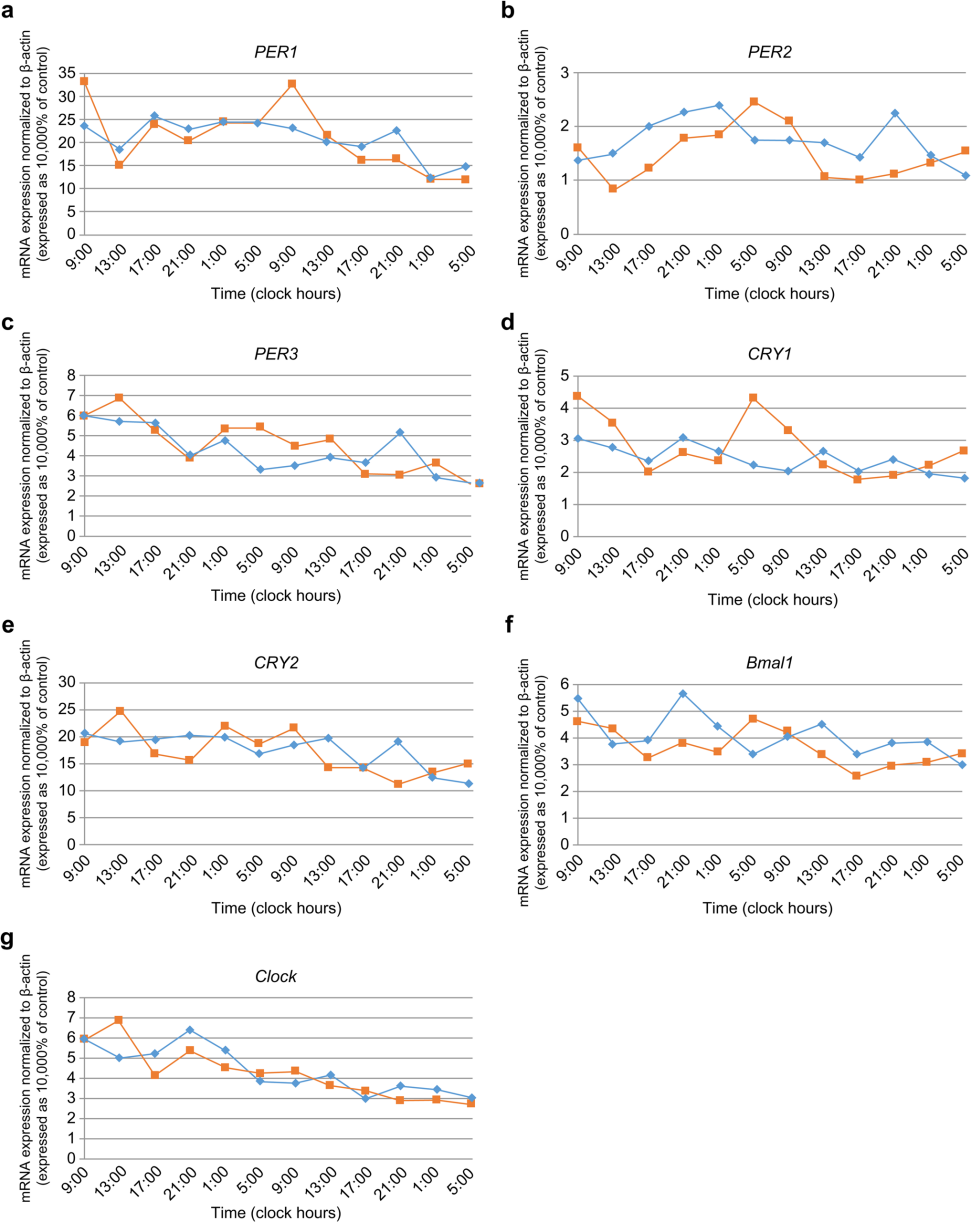
Time course-dependent changes in the relative messenger ribonucleic acid (mRNA) levels of **a** *Per1*, **b** *Per2*, **c** *Per3*, **d** *Cry1*, **e** *Cry2*, **f** *Bmal1*, and **g** *Clock* in peripheral blood leukocytes (PBL) from sham-operated (*n* = 7, yellow line) and acute subdural hematoma (ASDH) (*n* = 7, blue line) rats. mRNA levels are expressed relative to those of β-actin. Using the cosinor method analysis, the mRNA expression of *Per2*, *Cry1*, and *Bmal1* exhibited significant 24-h variation in sham-operated rats (*P* < 0.05). The standard error is not shown to ensure clarity of presentation

To determine the effects of ASDH on circadian rhythm regulation, we measured the mRNA expression of seven circadian rhythm-related genes in PBLs. The mRNA expression patterns of *Per1*, *Per2*, *Per3*, *Cry1*, *Cry2*, *Clock*, and *Bmal1* in PBLs of sham-operated rats (*n* = 7) and rats with ASDH (*n* = 7) were determined using real-time PCR (Fig. 1).

**Fig. 2:**
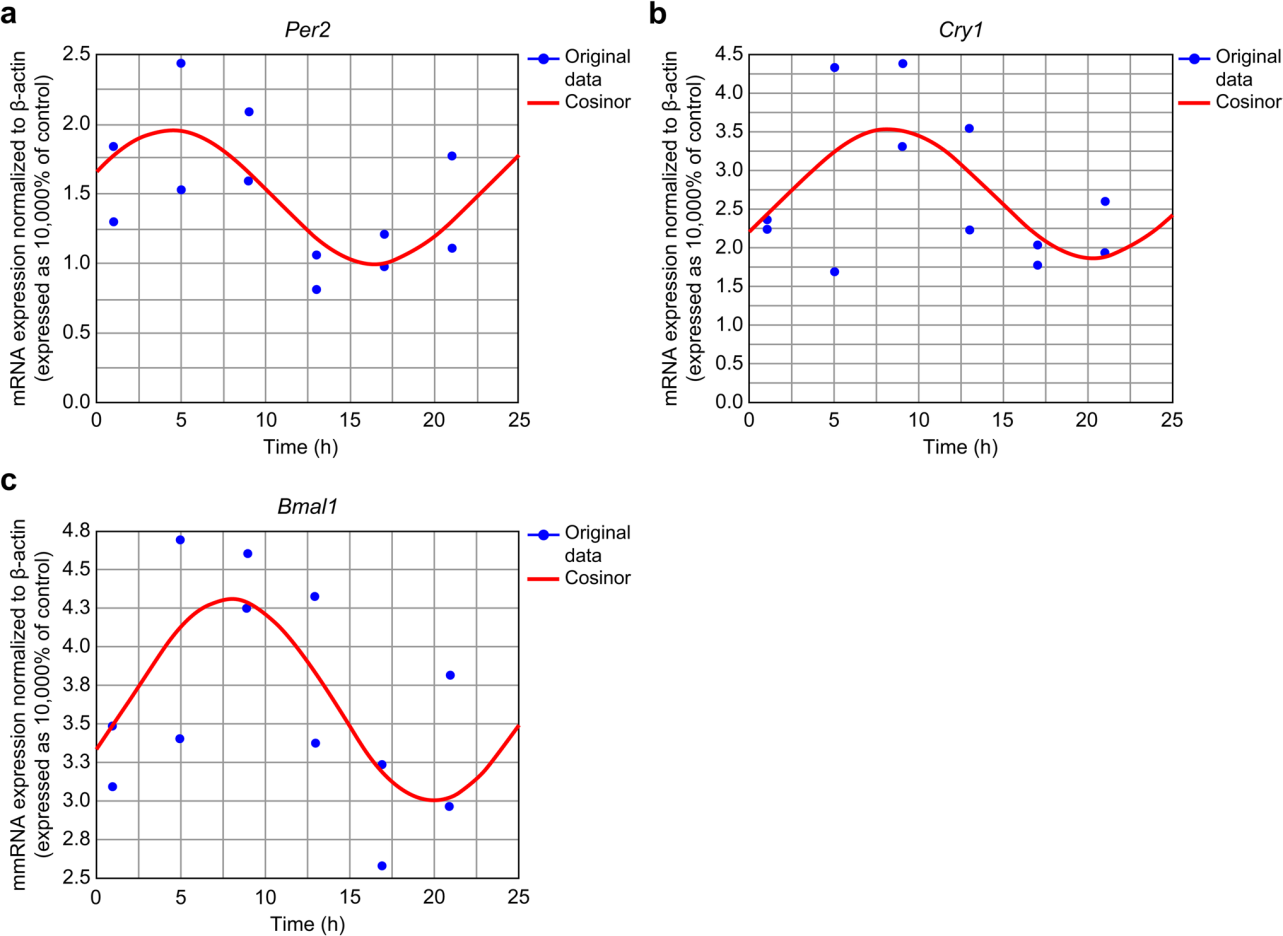
Time course-dependent changes in relative mRNA levels of **a** *Per2*, **b** *Cry1*, and **c** *Bmal1* in the PBLs of sham-operated rats. mRNA levels are expressed relative to those of β-actin. The estimated best-fit cosine curve (continuous line) is plotted by analyzing the mean expression in seven samples at each time point. For example, the two levels of expression at hour 1 represent the data obtained at the 1st and 25th h. The mRNA expression of *Per2*, *Cry1*, and *Bmal1* exhibited significant 24-h variations (*P* < 0.05)

The average mRNA expression at each time point was assessed using cosinor analysis. *Per2*, *Cry1*, and *Bmal1* mRNAs exhibited circadian expression rhythmicity in the PBLs of sham-operated rats (*P* < 0.05; Fig. 2). The acrophase of *Per2*, *Cry1*, and *Bmal1* mRNA expression in sham-operated rats was 4:26, 7:48, and 8:00, respectively (Table 2). No rhythmic expression was observed for *Per1*, *Per3*, *Cry2*, and *Clock* mRNAs in the PBLs of sham-operated rats. In ASDH rats, the rhythmic changes in the expression of *Per2*, *Cry1*, and *Bmal1* mRNA were disrupted. No rhythmicity was observed for *Per1*, *Per3*, *Cry2*, and *Clock* mRNAs in the PBLs of the ASDH rats.

**Table 2:** Rhythm features of messenger ribonucleic acid (mRNA) expression of circadian genes in sham-operated and acute subdural hematoma (ASDH) rats

	**Mesor**	**Amplitude**	**Acrophase (clock hours)**	***P*-value**
Sham-operated rats				
* Per1*	20.96	4.54	09:30	0.326
* Per2*	1.48	0.48	04:26	0.039 *
* Per3*	4.53	0.91	10:59	0.251
* Cry1*	2.79	0.94	07:48	0.017 *
* Cry2*	17.29	2.83	09:11	0.244
* Bmal1*	3.65	0.65	08:00	0.044 *
* Clock*	4.26	0.74	11:56	0.400
ASDH rats				
* Per1*	20.96	1.09	15:22	0.840
* Per2*	1.74	0.34	21:04	0.122
* Per3*	4.28	0.77	15:18	0.284
* Cry1*	2.44	0.17	15:31	0.662
* Cry2*	17.72	1.73	14:50	0.456
* Bmal1*	4.10	0.14	16.73	0.925
* Clock*	4.43	0.27	16.68	0.871

^*^P-value for statistical significance of parameter estimates: Rhythm detection was considered significant when *P* < 0.05, *ASDH* acute subdural hematoma

*Per1* period 1, *Per2* period 2, *Per3* period 3, *Cry1* cryptochrome 1, *Cry2* cryptochrome 2, *Bmal1* brain and muscle aryl hydrocarbon receptor nuclear translocator-like 1 *Clock* circadian locomotor output cycles kaput, ASDH acute subdural hematoma

### cBT analysis

**Fig. 3:**
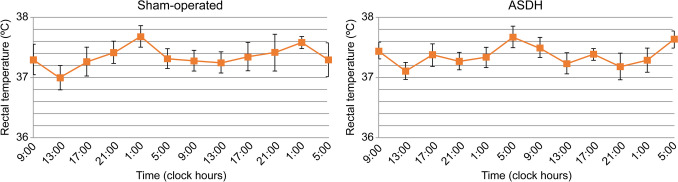
Time course-dependent changes in rectal temperature in sham-operated (*n* = 7) and acute subdural hematoma (ASDH) rats (*n* = 7). Data are expressed as mean ± standard error

**Fig. 4:**
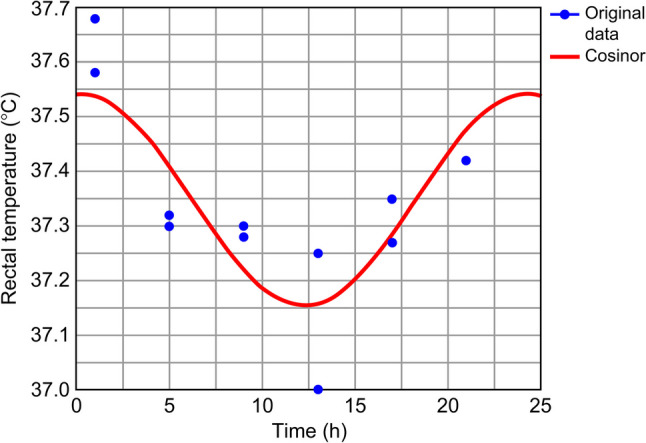
Time-related patterns of rectal temperature in sham-operated rats. *X*–*Y* plots represent the fitted cosine curves (continuous line) of rectal temperature measurements at 4-h intervals over 48 h. The curve is plotted by analyzing the measurements obtained for seven rats at each time point. For example, the two measurements at hour 1 represent the data obtained at the 1st and 25th h

**Fig. 5:**
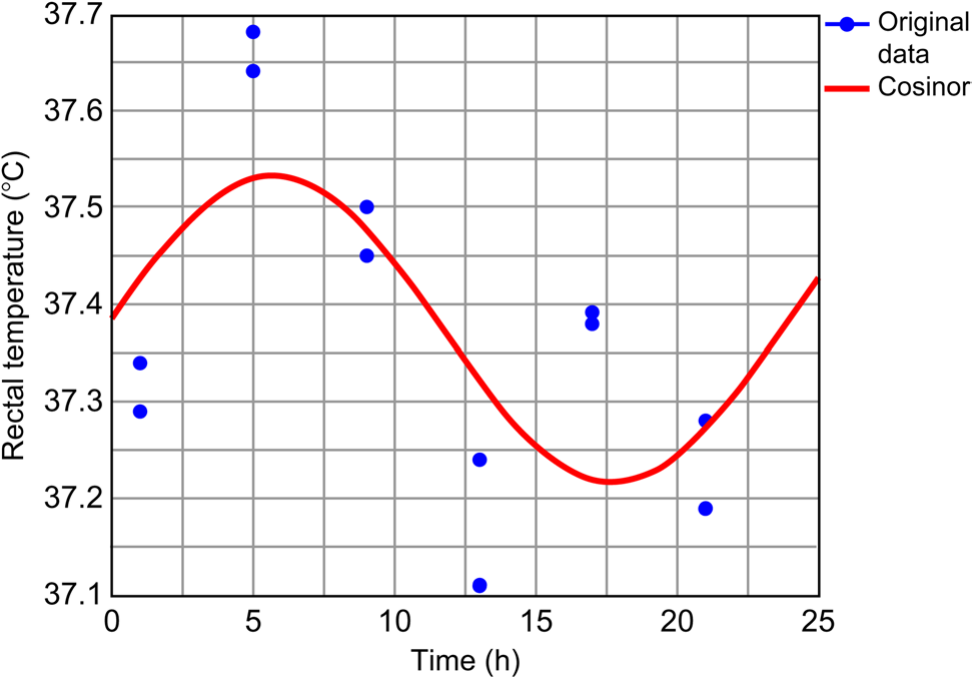
Time-related patterns of rectal temperature in ASDH rats. *X*–*Y* plots representing the fitted cosine curves (continuous line) of rectal temperature measurements at 4-h intervals over 48 h. The curve is plotted by analyzing the measurements for seven rats at each time point. For example, the two measurements at hour 1 represent the data obtained at the 1st and 25th h

To determine the effects of ASDH on the regulation of core body temperature, rectal temperature was measured using an anal probe every 4 h over 2 days (Fig. 3). The average rectal temperature at each time point was analyzed using cosinor analysis (Table 3). In the sham-operated rats, the temperature changes fit the 24-h day-night rhythm (*P* = 0.04), with the highest values occurring at 00:16 AM (Fig. 4). In the ASDH rats, the rhythmicity was disrupted (*P* = 0.06), with decreased amplitude and an acrophase shift to 05:42 (Fig. 5). The mesor was 37.35 °C in the sham-operated group and 37.37 °C in the ASDH group.

**Table 3:** Rhythm features of rectal temperature in sham-operated and acute subdural hematoma (ASDH) rats

	**Mesor**	**Amplitude**	**Acrophase**	***P-*value**
Sham-operated rats	37.35 °C	0.19	00:16	0.004*
ASDH rats	37.37 °C	0.16	05:42	0.063

*Statistically significant (*P* < 0.05)

*ASDH* acute subdural hematoma

## Discussion

This study is the first to determine the mRNA expression of circadian genes in PBLs in an ASDH animal model. We observed disrupted rhythmicity of *Per2*, *Cry1*, and *Bmal1* mRNA and rectal temperature after ASDH. Additionally, a depressed amplitude and acrophase shift were noted.

Oscillations in *Per1* and *Per2* mRNA expression were initially observed in cultured fibroblasts, suggesting the existence of peripheral circadian clocks [29, 30]. *Per* genes are involved in cell cycle and cancer development [31, 32]. SCN oscillations have since been detected in various cells outside the SCN as fluctuations in *Per1*, *Per2*, *Per3*, *Cry1*, *Cry2*, *Bmal1*, and *Clock* expression. The phase delay between SCN and peripheral expression is approximately 4–6 h [33]. Disrupted circadian function results in compromised adaptation to environmental changes, which is associated with various conditions in humans, including aging, neurological and psychiatric problems, metabolic disorders, reproductive abnormalities, and cancer development [8, 34]. SCN lesions cause dysregulation of peripheral oscillator rhythms [35] and altered gene expression in certain peripheral tissues [36]. An intrinsic transcriptional–translational feedback loop regulates rhythmic circadian gene expression [37]. The transcription factors, *Clock* and *Bmal1*, activate *Per* (*Per1*, *Per2*, and *Per3*) and *Cry* (*Cry1* and *Cry2*) genes by heterodimerizing in the nucleus and binding to response elements within respective promoter sequences. Furthermore, PER and CRY repressor proteins form cytoplasmic complexes, which are translocated to the nucleus to inhibit CLOCK/BMAL-mediated transcription [32, 38]. For a new cycle to begin, CRY proteins are targeted for proteasomal degradation through association with FBXL3 (a member of the F-box protein family) E3 ubiquitin ligase complexes [39–41]. This transcriptional–translational feedback loop imposes activating/repressive functions in an autoregulatory cyclic manner. In another circadian feedback loop, REV-ERB proteins, the members of the nuclear receptor superfamily of intracellular transcription factors, are activated by the CLOCK/BMAL dimer, which enters the nucleus to inhibit *Bmal1*. Therefore, *Per1* and *Per2* expression in rodent SCN peaks during the day, whereas *Bmal1* expression peaks at night [42–44]. These two feedback loops regulate circadian oscillations in gene expression.

Besides SCN, rhythmic circadian gene expression has been identified in the pineal gland, olfactory bulb, and forebrain [45–51]. Rhythmic circadian gene expression was detected in the lung, liver, stomach, adrenal glands, kidney, bone marrow, vasculature, adipose tissue, and peripheral blood in animals [42–44, 52–58]. Rhythmic *Per1*, *Cry1*, and *Bmal1* mRNA expression in human oral mucosa and *Per1* and *Bmal1* in human skin is known [33]. Rhythmic changes in *Per1*, *Per2*, *Per3*, *Cry1*, *Bmal1*, and *Clock* expression occur in the PBLs and whole blood cells in healthy human subjects [59–64]. These findings suggest specific expression patterns of circadian genes in various tissues and organs. However, previous studies have yielded controversial data regarding circadian expression rhythmicity and acrophase. Moreover, consistent conclusions regarding whether all circadian genes exhibit rhythmicity in peripheral blood cells are missing. Abnormal expression levels of Per2, Clock, and Bmal1 in oral mucosa and mononuclear cells at certain time points were detected in patients with sleep disorders after TBI [65]. Protein levels of some circadian genes have been quantified in peripheral tissues using western blot analysis to clarify their roles in various physiological and pathological conditions [66–69], but not in TBI models. This highlights the need for further research on circadian gene expression in numerous healthy individuals using standardized protocols and more frequent sample collections.

As circadian clocks are highly conserved across mammals, rodents represent valuable models for investigating the regulation of human circadian rhythms in many diseases [70, 71]. Similar mRNA expression rhythms were observed in human hepatoma cells and mouse liver, but differences in acrophase and amplitude existed [72]. Serial biopsies of human bone marrow and adipose tissue are performed for the time-series analysis of gene expression [53, 73], but it is difficult to perform for other human organs/tissues. Therefore, we adopted the ASDH rat model to explore the molecular mechanisms underlying ASDH-associated circadian clock dysregulation. PBLs were selected as source material to analyze circadian rhythm, considering the accessibility and animal welfare issues. We found that only *Per2*, *Cry1*, and *Bmal1* mRNA expression was rhythmic in sham-operated rat PBLs, but it was disrupted in ASDH rats, suggesting that the accumulation of blood in the subdural space with increased ICP is responsible for this change.

ASDH also disrupted the rhythm of rectal temperature, with reduced amplitude and a shift of the acrophase. The peak cBT in sham-operated rats was at 00:16 AM, which is comparable to the findings of a previous study [74]. In a study comprising 108 patients with moderate-to-severe TBI, 40.7% presented disrupted brain temperature rhythm; however, some patients with normal brain temperature rhythm still exhibited phase shifts [13]. Only a few studies have investigated the rhythms and acrophases of body or brain temperature in patients after brain injuries. Shifts in acrophase of cBT were observed in a study comprising 28 Alzheimer’s disease patients who presented with high nocturnal activity and fragmented sleep [75]. Among 100 patients with intracerebral hemorrhage, the rhythmicity of systolic blood pressure and heart rate was lost in 43% and 52% of patients, respectively [76]. In a study of 78 patients with basal ganglia hemorrhage after surgery, brain temperature remained intact in 55.1%, with acrophase shift in 60.3% of patients [77]. Furthermore, cosinor analysis of cBT in 86 patients with ruptured cerebral aneurysms revealed elevated mesors (37.8 ± 0.4 °C) with blunted amplitudes (0.27 ± 0.14 °C), and only 27% of acrophases remained within the normative 12–6 PM quadrant [78].

Traumatic brain injury (TBI) can lead to various forms of damage in the case of ASDH, encompassing localized cortical ischemic damage related to the hematoma, secondary injury due to increased intracranial pressure (ICP), and the release of toxic substances from the hematoma itself [79–81]. In their study using the high-frequency head impact (HFHI) and controlled cortical impact (CCI) mouse models of TBI, Korthas et al. revealed that different forms of brain trauma can result in distinct patterns of circadian and sleep disruptions [82]. Boone et al. observed that TBI disrupts the oscillation of *Per1* and *Bmal1* mRNA within the SCN and hippocampus [83]. Additionally, Li et al. found that, in context of TBI in rats, Bmal1 levels in the cerebral cortex decrease, exacerbating nerve damage by increasing the phosphorylation of P38 MAPK (mitogen-activated protein kinase) [84]. The effects of TBI on mRNA expression of peripheral oscillators are under-examined. Patients with TBI of varying severity may experience impaired circadian rhythm after injury to the hypothalamus. Changes in environmental synchronizers, such as ambient temperature in the ICU and feeding schedule, have been documented [85, 86]. Hemorrhage or other injuries of the retinohypothalamic tract may lead to the disruption of retinal inputs to the SCN [87]. Repeated impacts in high-frequency head impact (HFHI) mice have been demonstrated to result in the development of chronic inflammation and damage in the optic nerve/tract. Additionally, inflammation has been documented in the hypothalamus on the same side as the controlled cortical impact (CCI) injury in mice. [82]. In an autopsy study, microhemorrhage or ischemic necrosis in the hypothalamus was found in 42.5% of patients who died after severe TBI [88].

The increased ICP after ASDH in our model underlies SCN dysregulation. We injected nonheparinized blood into the subdural space to achieve an ICP of 22–25 mmHg. A larger blood injection and higher ICP may mimic the clinical scenarios in ASDH patients, wherein ICP impairs consciousness and causes neurological deficits. However, the animals may not survive through the entire study period owing to high ICP. As several factors may contribute to the dysregulation of circadian gene expression and temperature changes, further studies are required to explore other potential mechanisms.

Our study has a few limitations. First, the frequency and duration of longitudinal peripheral blood sampling were limited. Considering the welfare of animals and the possible effects of delayed brain ischemia and edema, we obtained 12 measurements over 2 days. Additional data collection may improve the reliability of cosinor analysis but may disturb the circadian rhythms of animals. Using telemetry devices may allow researchers to monitor the body temperature of animals continuously in their home environments without disturbance [17]. Second, unilateral limb weakness after ASDH makes it difficult to observe locomotor activity in animals. Third, our study exclusively employed male rats, which may limit the generalizability of the results to female rodents. Although the question of whether male or female rats exhibit higher variability in circadian rhythm study remains inconclusive, it is important to consider the effects of androgens and estrous cycle phase on the endogenous circadian period in male and female rodents, respectively. Fourth, the model using subdural blood injection does not entirely replicate the actual clinical scenarios of SDH, which are frequently associated with extensive primary brain injury. Further research is needed to investigate gene expression oscillations in SCN and different peripheral tissues and to explore the precise mechanisms underlying dysregulation of circadian gene expression after TBI, along with its relationship with body temperature changes.

In conclusion, this study provides novel insight into the rhythmicity of cBT and mRNA expression of circadian genes in PBLs in a rat model of ASDH. ASDH resulted in dysregulation of *Per2*, *Cry1*, and *Bmal1* mRNA expression in PBLs and rhythmic changes in body temperature during the first 48-h post-surgery. Although further studies are needed to explore the underlying molecular mechanisms, the dysregulation of mRNA expression may be targeted for the treatment of patients with post-TBI neurological and mental disorders.

## Data Availability

The datasets used and/or analyzed during the current study are available from the corresponding author on reasonable request.
